# Hepatico-renal Circulation

**Published:** 1851-09

**Authors:** 


*Hepatico-renal Circulation.*—M. Bernard has discovered the existence of a communication between the portal vein, the ascending cava and the kidneys, by means of which the urine is secreted from the blood which has not yet passed through the general circulation. This communication explains the rapidity with which substances taken into the stomach find their way into the urinary secretion, and also how it is that poisons taken into the alimentary canal are sometimes fatal and at others not, as well as why the presence of certain substances given to animals should be detected in the blood at one time and not at others According to his view "the renal veins have a double duty to perform; during the time of abstinence they conduct the return circulation from the kidneys; during digestion they act as arteries just as the pulmonary veins do for the lungs, and the portal vein for the liver. In effect, during the reflux of blood mentioned above there is a distinct pulsation perceivable in the cava and in the renal veins. This, though difficult to show, yet may be seen by killing a rabit during digestion and opening immediately the abdominal parietes.

"As M. Bernard remarked, when one reflects upon the matter, it is not so much to be wondered at, if we recollect that in fish and in reptiles there exists a porto-renal vein by which a certain quantity of blood passes directly to the kidneys from the mesenteric veins, only a portion being sent to the lungs."—*American Jour. Med. Sci.*, *July*, 1851.

